# Direct Electrosynthesis of an Amino Acid from a Biomass Derivative

**DOI:** 10.1021/acselectrochem.4c00171

**Published:** 2025-03-14

**Authors:** Zamaan Mukadam, Sihang Liu, Soren B. Scott, Yuxiang Zhou, Georg Kastlunger, Mary P. Ryan, Maria Magdalena Titirici, Ifan E. L. Stephens

**Affiliations:** † Department of Materials, Imperial College London, London SW7 2AZ, United Kingdom; ‡ Catalysis Theory Center, Department of Physics, Technical University of Denmark (DTU), 2800 Kgs. Lyngby, Denmark; § Department of Chemical Engineering, Imperial College London, London SW7 2AZ, United Kingdom; ∥ Department of Chemistry, University of Copenhagen, 2100 Copenhagen, Denmark

**Keywords:** Electrosynthesis, biomass, electrovalorization, amino acids, renewable energy

## Abstract

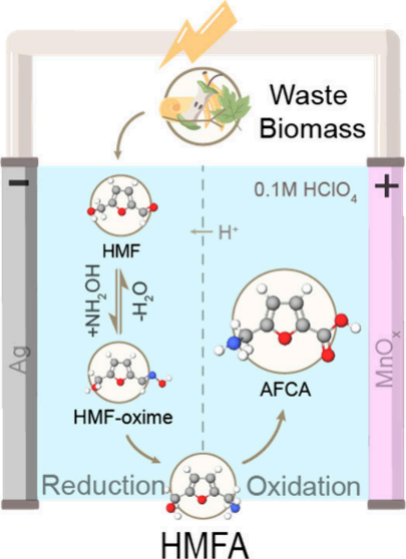

The electrochemical synthesis of nitrogen-containing molecules from biomass-derived compounds under ambient conditions is demonstrated, relying only on green sources of feedstock, renewable energy, and water. In this study, we report a two-step method of electrochemically synthesizing 5-(aminomethyl)furan-2-carboxylic acid (AFCA) from 5-hydroxymethylfurfural (HMF) using hydroxylamine (NH_2_OH) as the nitrogen source in an acidic electrolyte. In the first step, HMF was reductively aminated into (5-(aminomethyl)furan-2-yl)methanol (HMFA) using NH_2_OH as the source of nitrogen. This was followed by a second step, involving the oxidation of HMFA to AFCA on a manganese oxide (MnO_*x*_) anode at the same pH. MnO_*x*_ was able to selectively oxidize the alcohol group on HMFA to produce AFCA with 35% Faradaic efficiency without affecting the amine group. As both of these reactions are completed in a pH 1 electrolyte, it eliminates the need to separate HMFA before proceeding with the second reaction. Among different metal electrodes (Ag, Au, Cu, Pb, Pt and Sn) tested for the electrochemical reductive amination reaction, Ag electrodes displayed the best performance to selectively aminate HMF to the intermediate species, HMFA, with up to 69% Faradaic efficiency at mild potentials of −0.50 V_RHE_. Density functional theory calculations were carried out to explore a possible reaction pathway for the reductive amination on Ag(111), which suggests a thermodynamically feasible reaction even at 0 V_RHE_. The cathodic experimental reaction parameters were optimized to reveal that an electrolyte pH of 1 is optimal for the electrochemical reductive amination reaction. Our work shapes the future possibility of an electrochemical synthesis to produce AFCA without the need for any product separation between steps by combining the Ag cathode reaction to the MnO_*x*_ anode reaction sharing the same electrolyte. Since both the cathode and anode reactions both involve four electrons transferred, combining both half reactions in a single electrochemical reactor can eliminate the need for energy-wasting auxiliary counter reactions such as hydrogen evolution or water oxidation.

## Introduction

The global climate crisis has highlighted the need to move away from petrochemicals as our primary source of chemicals to avoid irreversible changes to the Earth’s climate. An alternative source of chemicals attracting interest is lignocellulosic biomass obtainable from agricultural waste.^1^ The acidic hydrolysis of cellulose and hemicellulose can yield furan containing chemicals such as furfural and 5-hydroxymethylfurfural (HMF),^2,3^ which are “platform chemicals”, meaning that they can be oxidized or reduced to produce a plethora of chemicals for use as fuels or in polymers.^4^ The alcohol and aldehyde side groups of HMF can be reduced, resulting in useful chemicals such as 2,5-bis(hydroxymethyl)furan (BHMF) and 2,5-dimethyfuran (DMF), with the latter having a high energy density comparable to that of fossil fuels.^5^ Further reduction of the aromatic furan ring can produce 2,5-bishydroxymethyltetrahydrofuran (BHMTHF), which is a vital solvent and fuel and has been demonstrated thermochemically using PdNi nanoalloys with H_2_ gas.^6^ Arguably, the most promising chemical obtainable from HMF oxidation is 2,5-furandicarboxylic acid (FDCA), which was stated as one of the most important chemicals for a sustainable future by the US Department of Energy,^7^ as it can act as a replacement monomer for terephthalic acid used in poly(ethylene terephthalate) (PET) polymers. At present, around 0.25 million metric tons of furfural is produced each year,^1^ and feasible ventures to upscale the production of HMF have yet to be identified. As lignocellulosic biomass is both abundant and a waste product, valorizing this biomass can act as an effective means to reduce our reliance on fossil fuels.

Amines are an important class of chemicals that are used in dye-stuffs, pharmaceuticals, polymers, and fertilizers.^8,9^ By incorporating nitrogen moieties into HMF molecules, it is possible to open up a wider variety of possible chemicals that have even more diverse uses compared to the standard HMF oxidation/reduction products. Aminating HMF would allow the production of chemicals such as 2,5-bis(aminomethyl)furan (DAMF), furfurylamine, (5-(aminomethyl)furan-2-yl)methanol (HMFA), and 5-(aminomethyl)furan-2-carboxylic acid (AFCA), the latter being an amino acid that can be sustainably sourced. These compounds can be used to produce polyamides, and some furanic amines have even shown therapeutic activity.^10,11^ AFCA is of particular interest as it can be self-polymerized into poly(AFCA), which has a structure analogous to that of Kevlar,^12^ but with a furan ring replacing a benzene ring. In addition, the permanent dipole and nonlinear nature of the furan ring compared to benzene can restrict ring rotation, allowing for greater thermal stability of the resulting polymer.^13,14^

The present industrial production of amines can involve using toxic cyanide as a source of nitrogen,^15^ which after nucleophilic addition on to a carbonyl group, requires a reduction using silanes^16^ or boranes^17^ to reduce the resulting nitrile group into an amine. This thermochemical amination requires high temperatures and pressures, and the reactions are usually carried out in organic solvents.^18^ Electrochemical reductive amination provides an attractive greener alternative for the amination of small biomass compounds by being able to complete the reaction in an aqueous electrolyte using non-toxic and non-flammable water as a proton source and electrons as reducing agents.^19−21^ In addition, electrochemical reductive amination reactions can eliminate the use of cyanide as a nucleophilic nitrogen source^22^ by using NH_3_ or hydroxylamine (NH_2_OH) instead. The carbon footprint of this process can be further lowered by producing NH_3_ using a greener Haber-Bosch process,^23^ and methods to produce NH_3_ through electrochemical nitrogen reduction are also being studied.^24,25^ NH_2_OH, meanwhile, can be produced electrochemically from waste NO_*x*_ products.^26^ Furthermore, the electricity needed to drive these reactions can be linked to renewable energy sources, resulting in the reactions being possible at room temperature and lower energy demands compared to thermochemical methods. Previous literature has demonstrated the electrochemical reductive amination using NH_2_OH for the synthesis of α-amino acids from pyruvic acid using TiO_2_ cathodes.^27^ Roylance and Choi further applied electrochemical reductive amination of furanic compounds using primary amine buffers in alkaline pH using Ag cathodes.^21^ In brief, this electrochemical reductive amination reaction involves the addition of a nucleophilic nitrogen source to a carbonyl group to form a carbonyl and imine/oxime equilibrium depending on the nitrogen source,^27^ removing a molecule of water in the process (Reaction 1a). This nitrogenated intermediate can then be directly electrochemically reduced on an electrode surface to an amine.
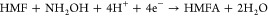
1
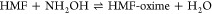
1a
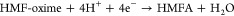
1b
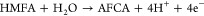
2

**Figure 1:**
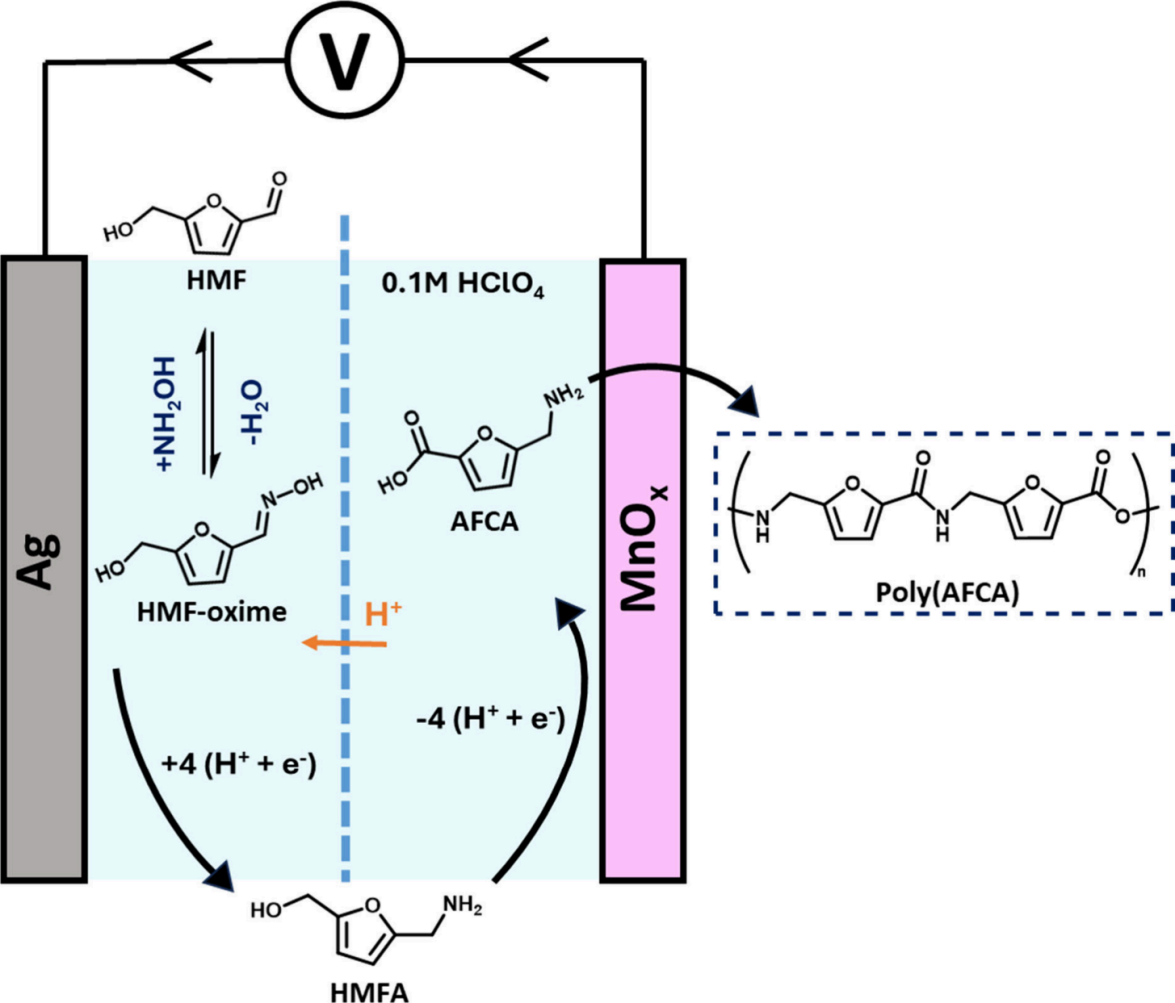
Proposed membrane separated electrochemical reductive amination of HMF to produce HMFA on Ag cathodes and the subsequent oxidation of HMFA into AFCA on MnO_*x*_ anodes in acidic media. AFCA is able to be self-polymerized into the renewable polyamide poly(AFCA).

Most industrial chemical syntheses require more than one step, which usually require reagent heavy chromatographic separation and purification of intermediates before each step, substantially increasing operation costs.^28,29^ Herein, this work demonstrates a method of electrochemically producing AFCA in two steps. First, the aldehyde group on HMF was reductively aminated into a primary amine, followed by an electrochemical oxidation of the C5 alcohol group into a carboxylic acid, with both reactions being demonstrated in an acidic pH 1 electrolyte. This factor will eliminate the need for any separation between the oxidation and reduction steps by utilizing the selectivity benefits of electrosynthesis. Furthermore, as the number of electrons transferred in the reduction and oxidation reactions are equal, the two half-reactions (Reactions 1 and 2 above) can be carried out on separate sides of a single electrochemical reactor (Figure 1), eliminating the need for auxiliary balancing reactions such as the hydrogen evolution reaction (HER) and the oxygen evolution reaction (OER). More specifically, Ag foil electrodes are used to reductively aminate HMF into HMFA in the presence of NH_2_OH as the external nitrogen source, which is followed by the selective oxidation of HMFA into AFCA by using manganese oxide (MnO_*x*_) sputtered onto carbon paper as the anode. MnO_*x*_ electrodes were chosen for the electrooxidation of HMFA, as it was shown in a previous study to selectively oxidize alcohols to acids.^30^ The reductive amination mechanisms on a model Ag(111) surface are explored by density functional theory calculations, and a thermodynamically favored pathway is proposed.

## Methods

### Materials

Perchloric acid (99.999%, Merck), 5-hydroxymethylfurfural (99%, Sigma-Aldrich), furfural (99%, Sigma-Aldrich), (5-(aminomethyl)furan-2-yl)methanol (95%, FluoroChem), hydroxylamine solution (50 wt % in H_2_O, 99.999% Sigma-Aldrich), carbon paper (Freudenberg H17, Fuel Cell Stores), silver foil (99.97%, GoodFellow), lead foil (99.99%, GoodFellow), gold foil (99.9%, GoodFellow), tin foil (99.95%, GoodFellow), copper foil (99.9%, Goodfellow), dimethyl sulfoxide (DMSO, analytical standard, Sigma-Aldrich), Nafion 117 membranes (Fuel Cell Stores), argon (99.999%, BOC), and Mn and Pt metal targets for sputter deposition (99%, AJA) were used.

### Electrode Preparation

Metal electrodes (1 × 1 cm^2^) were prepared by using fine sandpaper on the surface, followed by sonication in ethanol for 15 min and then rinsing with Milli-Q water (18.2 MΩ·cm) before use. 200 nm thick MnO_*x*_ and Pt electrodes were prepared using sputter deposition (AJA Magneton sputter deposition tool) onto a carbon paper substrate. For Pt deposition, the power was set at 30 W at 3 mTorr and an argon gas flow of 20 mL min^–1^. For MnO_*x*_ deposition, 200 W power was used at 3 mTorr with a gas flow of 20 mL min^–1^ argon and 5 mL min^–1^ O_2_. MnO_*x*_ anode production was confirmed using X-ray photoelectron spectroscopy (XPS, Thermo Fisher Kα high throughput system, 400 μm X-ray spot size using an Al Kα source) with spectra of the Mn 3s core level denoted in the Supporting Information. For scanning electron microscopy (SEM) images, Zeiss Auriga was used with the acceleration voltage of 5 kV, aperture size of 30 μm, and working distance of 5 mm. Energy dispersive X-ray (EDX) analysis was also performed on the same machine with the acceleration voltage of 20 kV, aperture size of 60 μm, and working distance of 10 mm.

### Electrochemical Experiments

Electrochemical experiments were carried out in a custom made three compartment H-cell (Cambridge Glassware) separated with a Nafion 117 membrane using a potentiostat (Ivium Vertex One) to control the applied potential. The electrolyte was purged using argon gas for 10 min before any experiments to remove any dissolved oxygen and was not purged with gas during any experiment. Linear sweep voltammograms (LSVs) were performed in a 0.1 M HClO_4_ solution (pH 1) prepared in Milli-Q water and swept in a cathodic direction at a scan rate of 50 mV s^–1^ for reductive amination experiments and swept in an anodic direction at the same scan rate for electrooxidation experiments. All potential values were *iR* corrected using electrochemical impedance spectroscopy (EIS) to obtain the uncompensated resistance values before any electrochemical experiments. 41 frequencies were used starting at 10,000 Hz and ending at 1 Hz with an amplitude of 0.03 V and were taken before each experiment at 0 V_RHE_. The values of the uncompensated resistance remained constant at around 5–10 Ω for each experiment in 0.1 M HClO_4_ (Figure S7) and were automatically compensated for at 85% using the Ivium software. Gold mesh was used as the counter electrode, and a saturated Hg/HgSO_4_ electrode (SI Analytics) was used as the reference. The reference electrode was calibrated against a homemade reversible hydrogen electrode (RHE), and all measurements herein are reported on the RHE scale with the conversions carried out using eq 3.
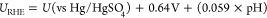
3

### Product Analysis

Aliquots were taken directly after electrolysis experiments with no deuterated solvent added for quantitative analysis, which was performed using quantitative nuclear magnetic resonance (qNMR, Bruker Av400 spectrometer) with 5 mM DMSO as an internal standard. ^1^H Nuclear Overhauser Effect Spectroscopy (NOESY) was used to suppress the water peak to enable identification of any peaks of interest between 4 and 5 ppm. The presence of the aminated furanic compounds was confirmed by comparing NMR spectra of previous studies for their synthesis.^31,32^ The Faradaic efficiency of detected products was then calculated using eq 4.1, where *n*, *z*, *Q*, and *F* are the moles, electrons transferred per mole, total charge passed, and the Faraday constant (96485 C mol^–1^), respectively. The Faradaic efficiency was calculated after 3 h for each experiment. The selectivity of the biomass products was calculated using eq 4.2.
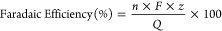
4.1
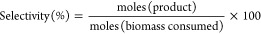
4.2

### Computational Details

Density functional theory calculations were conducted using the Vienna *ab Initio* Software Package (VASP).^33^ Core electrons were described by projector augmented wave (PAW) potentials.^34^ Valence electrons were described by plane waves with a kinetic energy up to 400 eV. The RPBE functional^35^ was used and complemented by Grimme’s D3^36^ to better describe the non-covalent interaction of the large organic reaction intermediates with surfaces. The lattice constant for *fcc*-Ag was identified as 4.16 Å in reasonable agreement with the experimental value.^37^ A 3 × 3 × 1 k-point mesh was applied for the 4 × 4 × 4 Ag(111) supercell applied in the simulations with the bottom two layers fixed to the bulk structure. All geometries were optimized until forces were less than 0.02 eV Å^–1^. A series of adsorption configurations was screened, and the most stable was included in the free energy diagram. The ASE Thermochemistry class^38^ was used to determine the Helmholtz and Gibbs free energies within the harmonic and ideal gas limit for adsorbed and gaseous species (H_2_, HMF, NH_2_OH, and HMFA), respectively, at 300 K. For simplicity, we did not consider the solvation of the intermediates in this work, which requires extensive benchmarking work. The effect of the applied potential on energetics was included based on the computational hydrogen electrode (CHE) model introduced by Nørskov et al.^39^ Here, we take advantage of the equilibrium of the proton–electron pair (H^+^ + e^–^) and gaseous H_2_ at standard conditions and 0 V_RHE_ and the potential dependence of the electronic chemical potential is accounted for by a linear term. Thus, the chemical potential of the proton–electron pair is substituted following the relation:
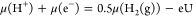
5

## Results and Discussion

The first step of the electrochemical reductive amination of the HMF reaction involves a nucleophilic attack by the nitrogen source across the aldehyde group, removing water in the process. NH_2_OH was chosen over NH_3_ as the nitrogen source for all amination reactions due to its enhanced nucleophilic activity as a consequence of the α-effect.^40^ This resulted in only a 1:1 equiv of NH_2_OH to carbonyl bond being needed to convert nearly all of a 20 mM solution of furfural into oximes (Figure S4), as expected from the stoichiometry. On the other hand, we observed incomplete conversion of aldehydes to imines by NH_3_ even when high concentrations of NH_3_ were used, in agreement with previous literature.^27,41^ When NH_2_OH is used as the nitrogen source, an equilibrium between HMF and the oxime intermediate is formed, which needs to be pushed towards the oxime for subsequent reduction; otherwise, unwanted aldehyde reduction is possible (eq 6.4). Furthermore, the electrochemical reductive amination reaction has other competing reactions, which need to be minimized to increase electrochemical efficiency, such as the hydrogen evolution reaction (HER, eq 6.1) and the reduction of NH_2_OH into NH_3_ (eq 6.2). Using a 1:1 equiv of NH_2_OH to HMF should, in principle, solve the issue of NH_2_OH reduction as a competing reaction, as in theory, no NH_2_OH will remain in solution after the complete substitution of the carbonyl bonds of HMF. We derived the equilibrium potentials versus the RHE for the major reduction reactions in this work based on DFT calculations of stable gases and liquids at 298.15 K and 1 bar:
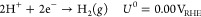
6.1
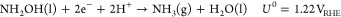
6.2
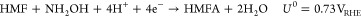
6.3
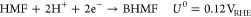
6.4

### Screening of Suitable Electrodes for Reductive Amination

**Figure 2:**
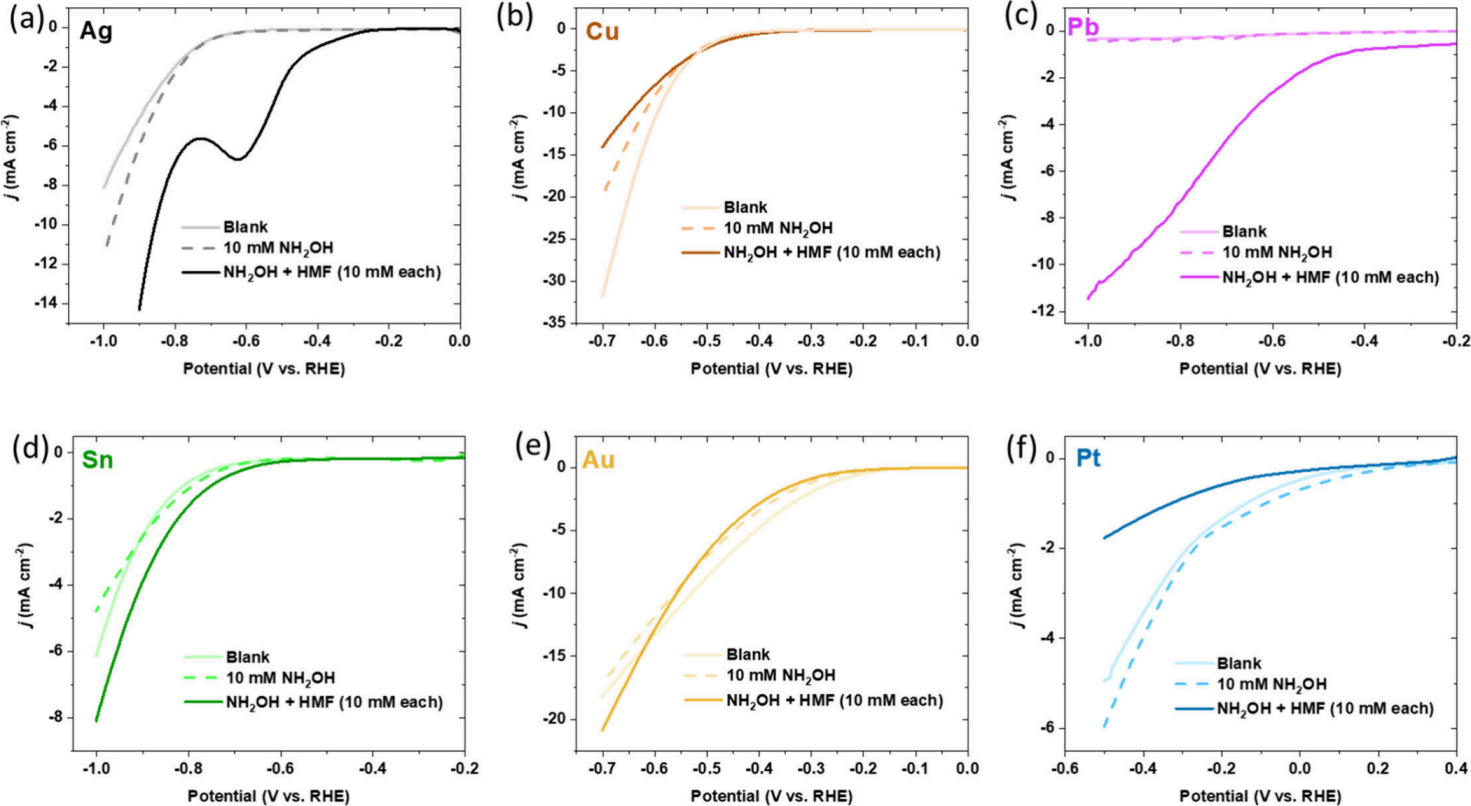
Linear sweep voltammograms (in the cathodic direction) of 10 mM HMF in the presence of 10 mM NH_2_OH on (a) Ag, (b) Cu, (c) Pb, (d) Sn, (e) Au, and (f) Pt electrodes. Reaction conditions: 0.1 M HClO_4_ electrolyte (pH 1); scan rate of 50 mV s^–1^ at room temperature. Each LSV was the second measured scan.

Previous studies on electrochemical reductive amination used ammonium-based buffer solutions as the electrolyte (pH 10–11).^20^ A high concentration ammonium buffer ensures that the imine intermediate formation is favored, but this limits the reaction conditions to alkaline electrolytes (Table S1). In contrast, using a more powerful nucleophile, such as NH_2_OH, allows us to operate in acidic electrolytes and avoid the pH constraints imposed by buffering systems. Various metal surfaces were tested for the reductive amination of 10 mM HMF in a 0.1 M HClO_4_ (pH 1) electrolyte. A low pH was chosen, as previous studies for electrochemical reduction reactions of furanic compounds preferred acidic electrolytes for more efficient synthesis.^42^Figure 2 shows the LSVs of Ag, Cu, Pb, Sn, Au, and Pt electrodes tested for HMF reductive amination. Ag^43^ and Cu^42^ were chosen because they showed activity for furfural/HMF reduction in acidic media in previous studies, and Pb was chosen for its high overpotential for the HER, with us hypothesizing biomass will be adsorbed onto the surface, preferably alongside some protons for subsequent reduction reactions. Similarly, Sn was chosen for its relatively high affinity for oxygen.^44^ Au and Pt were chosen as controls as they are active for a number of electrooxidation applications including alcohol fuel cells.^45,46^ For each electrode, a cathodic blank linear sweep voltammogram (LSV) of the electrolyte was taken, for which the increase in current density is attributed to the HER. This was followed by a cathodic sweep after the addition of 10 mM NH_2_OH, for which this increase in current density was associated with NH_2_OH reduction, subsequently followed by a cathodic sweep after the addition of 10 mM HMF after leaving it to stir for 10 min to allow enough time for NH_2_OH addition onto the carbonyl bonds. We observed that after just 10 min of vigorous stirring almost all of the carbonyl bonds in the biomass reacted with the hydroxylamine (Figure S4). For a scale-up that uses higher concentrations of carbonyl-containing biomass, it would be useful to use a slightly higher molar equivalent, e.g., 1:1.2 ratio, and increase the stirring time as well as confirm no more carbonyl bonds are present using NMR. The increase in current density during this LSV is attributed to the reductive amination of the HMF-oxime intermediate into HMFA.

The Pt and Au control electrodes were determined to be unsuitable for the electrochemical reductive amination reaction due to the HER and NH_2_OH reduction onset potentials (defined in this study as the potential required to achieve a −1 mA cm^–2^ current density) being more anodic than the biomass reduction onset, suggesting that the HER is likely to dominate before any biomass reduction. This contrasted with the biomass reduction LSVs for Cu, Sn, Ag, and Pb electrodes, which all exhibited a more anodic onset potential than the HER, suggesting biomass reduction was favored. The difference in onset potential was most apparent on Pb and Ag electrodes, with a cathodic peak putatively due to mass transport on Ag at around −0.60 V_RHE_. We deemed Cu electrodes unsuitable for reductive amination as the onset potentials for the HER and biomass reduction were too close together. In addition, Cu electrodes have been reported to deoxygenate furfural in acidic pH, which can cause selectivity issues.^47^ Sn electrodes displayed a more cathodic potential for biomass reduction compared to both Ag and Pb, and Pb electrodes displayed a lower current density at less cathodic potentials for the biomass reduction and have issues with toxicity (Figure 2c). We also tested each electrode in constant potential experiments at −0.50 V_RHE_ for 3 h and recorded the Faradaic efficiency of the HMFA produced. We observed that Ag electrodes displayed the highest Faradaic efficiency (∼69%), with the next highest being Cu electrodes at only 56% (Figure S8). As predicted, the HER dominated on Pt and Au electrodes, where ∼1% Faradaic efficiency for HMFA production was observed. For this reason, Ag electrodes were chosen for further optimization and mechanistic understanding due to their apparent enhanced activity for amination compared to the rest.

### Theoretical Understanding of HMF Reductive Amination on Ag(111)

**Figure 3:**
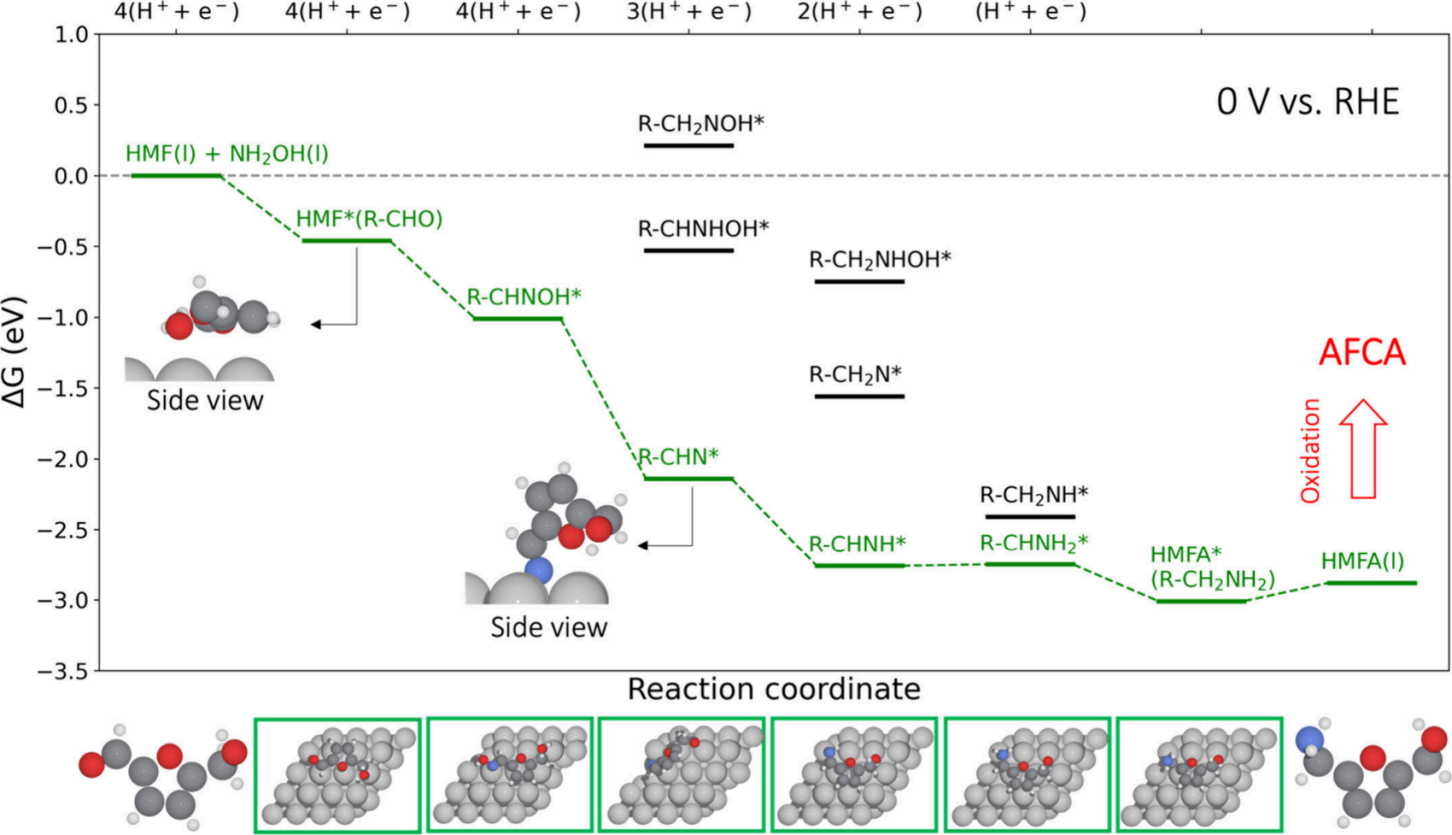
Calculated free energy diagram of HMF reductive amination towards HMFA over the Ag(111) surface at 0 V vs. RHE. To focus on the reducible −CHO group in HMF, we denote HMF as R-CHO with R representing the unreacted “CH_2_OH-furan-” group in the furanic compound. The thermodynamically favored pathway is colored in green, while the parallel but less favored states are in black. The side view for HMF* (R-CHO) and R-CHN* are shown as insets, with the other optimized structures summarized in Figure S1. Color codes: light gray, hydrogen (H); dark gray, carbon (C); red, oxygen (O); blue, nitrogen (N); gray, silver (Ag).

To unveil the origin of the high activity on Ag electrodes, we explored the HMF reductive amination mechanism on an Ag electrode (modeled as Ag(111) surface) using density functional theory (DFT); these results are shown in Figure 3. HMF tends to bind on Ag(111) flatly, with the −OH group being chemisorbed on the surface, as reported previously.^48^ At 0 V_RHE_, the pathway of HMF* → R-CHNOH* → R-CHN* → R-CHNH* → R-CHNH_2_* → HMFA* is thermodynamically facile with the last protonation step, i.e., R-CHNH* → R-CHNH_2_* being slightly endergonic (0.02 eV). The key intermediate R-CHN* consists of N being bound in a surface hollow site, while the furanic backbone is desorbed. In contrast, the other two reaction pathways via R-CH_2_NOH* or R-CHNHOH* are limited by the formation of R-CH_2_NOH* or R-CHNHOH* from R-CHNOH*, respectively. In short, the facile thermodynamics of HMF reductive amination makes Ag a potentially good electrocatalyst to produce HMFA even at 0 V_RHE_.

### Parameters Affecting Reductive Amination on Ag

**Figure 4:**
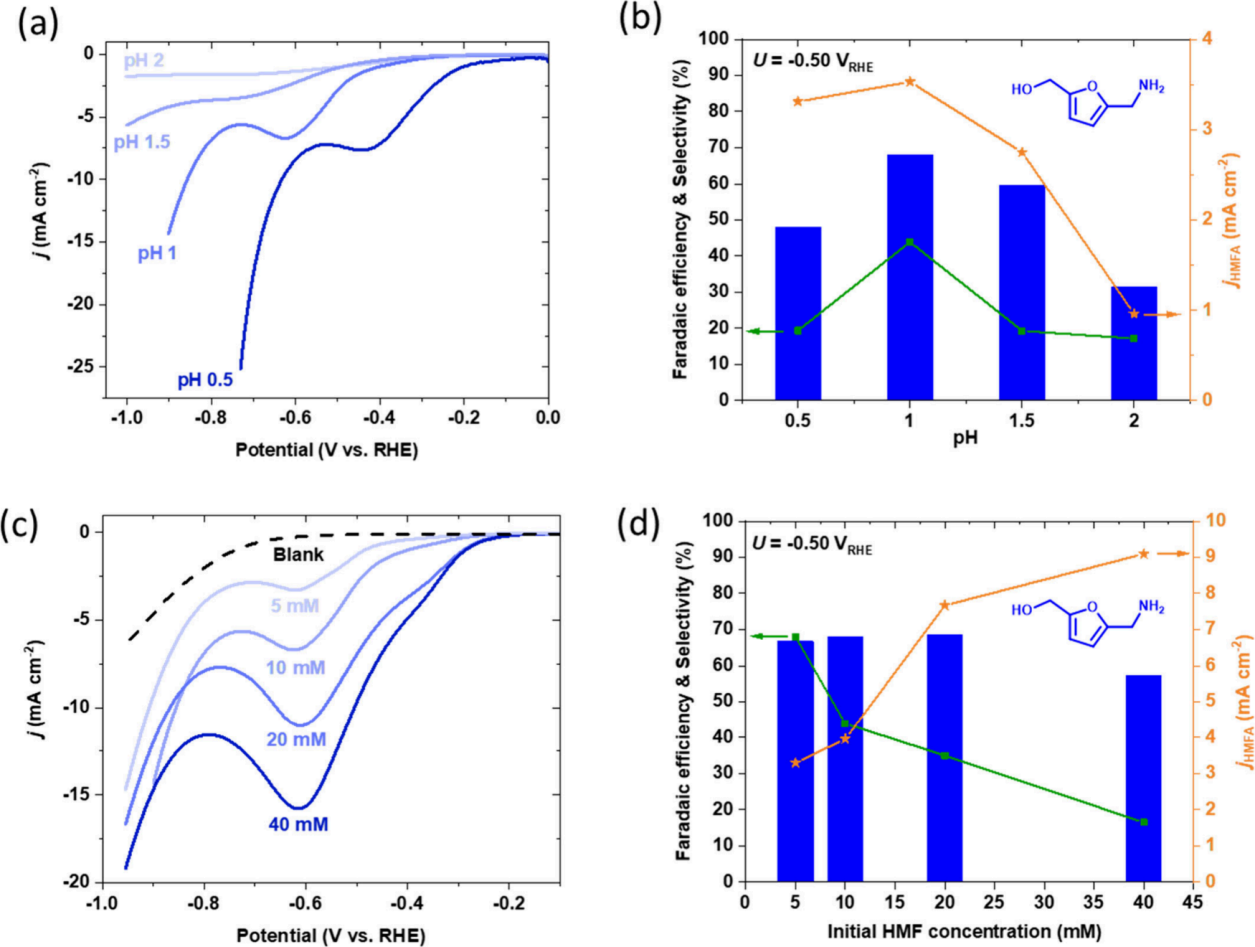
Electrochemical reductive amination of HMF into HMFA on Ag electrodes. (a) LSVs, (b) Faradaic efficiencies, partial current densities, and selectivity of HMF reductive amination at differing acidic pH values (0.5, 1.0, 1.5, and 2.0). (c) LSVs, (d) Faradaic efficiencies, partial current densities, and selectivity of HMF reductive amination at differing initial concentrations of HMF and NH_2_OH (0, 5, 10, 20, and 40 mM for both). Reaction conditions: HClO_4_ electrolyte solution adjusted for specific pH; scan rate for LSVs was 50 mV s^–1^ in the cathodic direction, and all constant potential experiments were done at −0.50 V_RHE_ for 3 h. The HMF and NH_2_OH concentrations for the reaction in (a) and (b) were 10 mM.

The effects of pH and the initial biomass concentration on the rates of HMF reductive amination were studied on Ag electrodes. It is likely that the active species for the amination reaction is the protonated oxime intermediate, which can have an increased tendency to adsorb onto the cathode. Figure 4a,b shows LSVs of HMF reductive amination at varying acidic pH and the corresponding Faradaic efficiencies, partial current densities, and selectivities of HMFA obtained at −0.50 V_RHE_ after 3 h. As the pH decreases, the cathodic peak, which is assumed to be associated with mass transport limitation, shifts more anodically while the total current density increases. This suggests that a lower pH increases the reductive amination activity, which could imply that the protonation of the oxime (reaction below) is important. However, Faradaic efficiencies and partial current densities of HMFA are highest at pH 1 and decrease at both lower and higher pH. We attribute this to increased rates of HER at pH 0.5 and lower rates of oxime protonation and subsequent surface adsorption at pH 1.5 and 2. The p*K*_a_ of the oxime protonation (estimated to be around 1–1.3)^49^ could be significant as a pH 1 electrolyte would mean almost all the oximes are protonated, which can make them likelier to adsorb onto the electrode surface. This draws similarities to a study by Kwon et al.^50^ who suggested that, for the electrooxidation of alcohols, the highest reaction rate is at the pH close to the p*K*_a_ of the reacting species. The selectivity of HMFA is also the highest at pH 1. However, it is still relatively low at 44% with the missing carbon balance likely raising HMF degradation at low pH.^51,52^
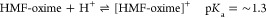


The effect of the initial concentrations of HMF and NH_2_OH was also tested to achieve the optimal conditions for HMF amination on Ag foil, including minimal mass transport limitations. Very high concentrations of HMF may be detrimental to product selectivity as the molecules can degrade into humin side products,^53^ of which the insoluble humins may deposit on the Ag surface negating electrochemical reduction activity. Figure 4c shows that increasing concentrations of HMF and NH_2_OH coincide with increases in the total current density likely due to an increase in concentration of protonated oximes available. The Faradaic efficiency of HMFA production after 3 h of constant potential electrolysis at −0.50 V_RHE_ is relatively independent of the initial concentration of HMF and NH_2_OH in the range of 5–20 mM initial concentration, with a slight drop in Faradaic efficiency at 40 mM HMF + NH_2_OH (Figure 4d). On the other hand, selectivity falls much faster as the starting concentration increases. We postulate this drop in selectivity is probably due to the formation of humin side products which are more likely to occur at high concentrations due to the acid-catalyzed condensation reactions. This was supported by the selectivity steadily decreasing as the concentration of HMF increased and more unidentifiable peaks apparent in the relative NMR spectra. A higher selectivity was achieved at lower concentrations likely due to a slower rate of self-condensation reactions between HMF molecules. Interestingly, the partial current density of HMFA production shows a rather linear increase from 5 to 20 mM HMF and starts to steadily decrease at higher concentrations, signaling that, under these conditions, about 20 mM HMF can be reductively aminated on Ag without too many mass transport limitations. Furthermore, with increasing biomass concentration, the selectivity drop is much more significant than the Faradaic efficiency drop signaling that humin formation requires fewer electrons than reductive amination, consequently forming radical species because of single-electron transfers, which may result in the formation of long chain oligomers.

### HMFA Oxidation on MnO_*x*_

**Figure 5:**
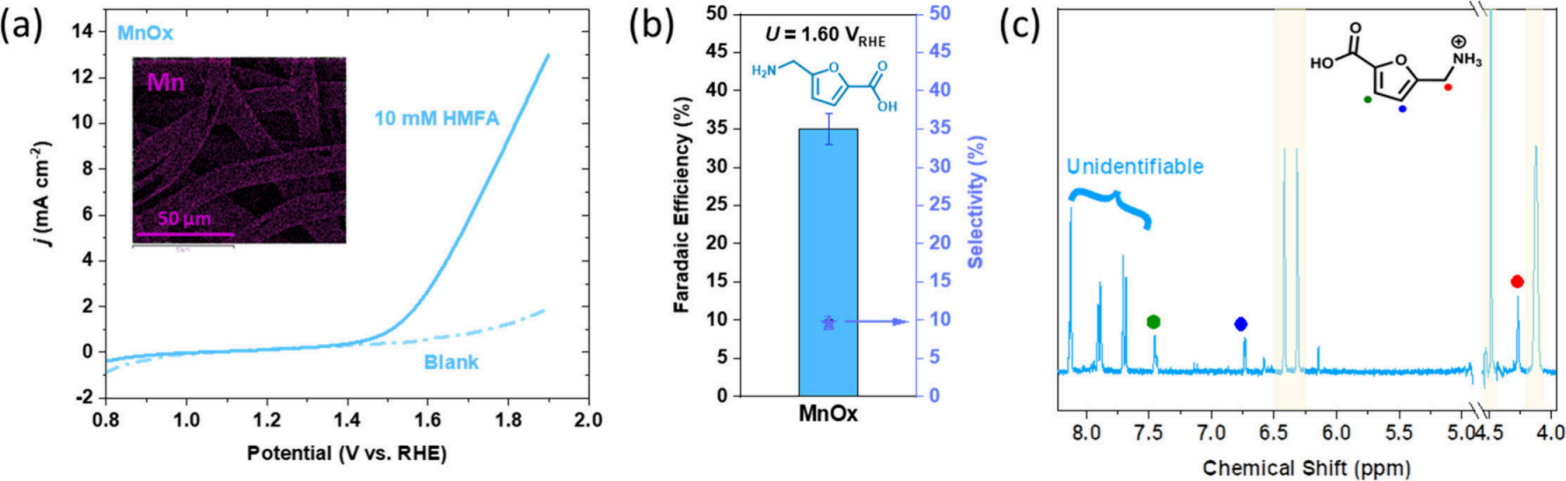
Electrochemical oxidation of HMFA using 200 nm MnO_*x*_ electrodes sputtered onto Freudenberg H17 carbon paper. (a) LSVs of MnO_*x*_ electrodes in pH 1 electrolyte before (“blank”, dashed line) and after (solid line) the addition of 10 mM HMFA. Inset depicts EDX image of Mn in a sputtered MnO_*x*_ on carbon electrode. (b) Faradaic efficiencies of detected liquid products after constant potential experiments at 1.60 V_RHE_ for 20 h. Error bars were produced after two separate experiments. Reaction conditions: 0.1 M HClO_4_ electrolyte (pH 1); scan rate for LSVs was 50 mV s^–1^. (c) ^1^H NMR spectra of AFCA produced after electrolysis; yellow highlighted peaks represent HMFA. Full NMR spectra with integration values are shown in the Supporting Information.

MnO_*x*_ anodes have been previously reported to oxidize HMF into FDCA in acidic electrolytes in previous studies,^30^ specifically using MnO_*x*_ electrodeposited onto fluorine-doped tin oxide (FTO). Thus, MnO_*x*_ was tested for its oxidation ability to selectively oxidize the primary alcohol group of HMFA into a carboxylic acid. MnO_*x*_ was sputter deposited onto carbon paper and characterized using XPS (Figure S5) with the main oxidation state that was detected being Mn^4+^. Figure 5a shows the LSV of a pH 1 HClO_4_ electrolyte before and after the addition of 10 mM HMFA oxidation using the MnO_*x*_ anode as the working electrode. We attributed any current above 1.40 V_RHE_ in the absence of HMFA, to water oxidation. In the presence of 10 mM HMFA, a much more substantial current is sustained over the same range, which we attribute to HMFA oxidation. AFCA was detected in the electrolyte using NMR spectroscopy following chronoamperometry experiments at 1.60 V_RHE_, and the calculated Faradaic efficiency was 35%. Whilst successful −CH_2_OH oxidation was observed using MnO_*x*_, a large amount of missing charge was unaccounted for, likely a combination of some water oxidation, carbon corrosion, and HMF degradation into humin side products evidenced by unidentifiable peaks in the ^1^H NMR spectra (Figure 5c) and a low selectivity of 9.65%. These unidentifiable side products for HMF oxidation in acidic media were also reported by Kubota and Choi^30^ with relatively low selectivities and a maximum Faradaic efficiency of 58% when using MnO_*x*_ annealed onto FTO glass. This is not much of a surprise as furanic compounds are notoriously unstable in acidic media and have many degradation routes including oligomer formation and ring-opening reactions.^54,55^ The chronoamperometry of MnO_*x*_ shows a progressive decrease in current density over time (Figure S9), likely a result of HMFA consumption and catalyst degradation. XPS and EDX analysis of the MnO_*x*_ anode post-chronoamperometry showed significant Mn removal from the electrode surface after 20 h (Figure S6 and Figure S10), suggesting that MnO_*x*_ is relatively unstable in acidic media during oxidation reactions likely because of Mn dissolution. Mn dissolution from sputtered MnO_*x*_ electrodes at similar conditions was also seen in a separate study by Frydendal et al.,^56^ in which complete Mn dissolution was reported in just a few hours. Whilst displaying a relatively low selectivity and Faradaic efficiency, what is significant here is the fact that MnO_*x*_ was able to selectively oxidize the alcohol group in HMFA to a carboxylic acid without affecting the primary amine group. It could be argued that using a support more robust than carbon paper alongside some thermal treatment could help with greater long-term electrode stability, but this optimization is out of the scope of this present work.

In principle, by combining the anodic and cathodic reactions together, we could achieve an electrochemical synthesis of AFCA without the need to separate HMFA before commencing the next reaction. This synthesis can be selective toward producing AFCA as the reduction and oxidation reactions do not interfere with the functional groups for which each electrode is responsible. For the oxidation reaction, this is likely due to the primary amine being in its protonated form, making adsorption to the anode more difficult. This is a reasonable assumption as aqueous amine oxidation has been reported in prior studies and requires high potentials in strongly alkaline electrolytes.^57^ In a batch reactor like we demonstrate here, the synthesis would need to happen sequentially: (i) first reductively aminate HMF into HMFA using Ag cathodes to produce as much HMFA as possible and then (ii) to switch electrodes to anodically oxidize HMFA into AFCA using MnO_*x*_. It is important for the amination reaction to happen first as alcohol oxidation on MnO_*x*_ is unselective and will oxidize both the C2 and C5 substituents, resulting in FDCA as the main product.^30^ Another important factor that we should note is that the amination (specifically the reduction of the oxime) should be completed to near 100% conversion before any oxidation. This is to avoid any irreversible electrochemical oxidation of the oxime into nitro groups, which reduces the selectivity of AFCA production. For this reason, the reduction and oxidation cannot happen “in the same pot”, but must at least be separated by a membrane, motivating the two-pass electrochemical reactor envisioned in Figure 1.

## Conclusions

In this contribution, we conceptualized a two-step electrosynthesis of a furanic amino acid directly from HMF, a biomass-derived chemical, with the possibility of having no separation steps in between reactions and a two-pass reactor that uses the electrons from the oxidation to drive the reduction, avoiding energy-wasting auxiliary half-reactions. The aldehyde group on HMF was able to be reductively aminated on Ag foil cathodes using NH_2_OH as the nitrogen source to produce HMFA with up to 72% Faradaic efficiency in an optimal pH 1 HClO_4_ electrolyte at −0.50 V_RHE_. DFT calculations also show a facile thermodynamic process for reductive amination on Ag even at 0 V_RHE_. HMFA was further electrochemically oxidized on a sputtered MnO_*x*_ anode to produce the amino acid, AFCA, with 35% Faradaic efficiency. Whilst we have proven the concept of a possible one-pot synthesis of AFCA in the future, the MnO_*x*_ sputtered onto carbon paper was unstable and significant amounts of Mn dissolution were apparent, alongside low selectivities likely resulting from humin production. Future studies from this work will entail methods of making sure MnO_*x*_ can be reasonably stable for this reaction, which can also be trialed in a continuous electrochemical flow cell. Research efforts will go into finding methods to lower the mass transport limitations of each half reaction in a future membrane separated flow cell, which will be supported by kinetic studies to elucidate the most optimal parameters. This work realizes room-temperature electrosynthesis of value-added amino acids using biomass derivatives, opening new avenues for functionalizing furanic compounds without cyanide or in organic solvents.

## Supporting Information Available

Additional characterization, analysis, and electrochemical data (PDF)
