# Supplementary material for: Direct Electrosynthesis of an Amino Acid from a Biomass Derivative
Source: ACS Electrochem. 2025 Mar 14;1(5):699–708. doi: 10.1021/acselectrochem.4c00171 (PMC12051197; doi:10.1021/acselectrochem.4c00171)
Supplement: Supplementary file 1 — ec4c00171_si_001.pdf [file ec4c00171_si_001.pdf]

Supporting Information

Direct Electrosynthesis of an Amino Acid from a Biomass Derivative

Zamaan Mukadam[1], Sihang Liu[2], Soren B. Scott[1,4], Yuxiang Zhou[1], Georg Kastlunger[2], Mary P. Ryan[1], Maria Magdalena Titirici[3], Ifan E. L. Stephens[1]*

Affiliations:

[1]Department of Materials, Imperial College London, London, UK, SW7 2AZ.

[2]Catalysis Theory Center, Department of Physics, Technical University of Denmark (DTU), 2800 Kgs. Lyngby, Denmark.

[3]Department of Chemical Engineering, Imperial College London, London, UK, SW7 2AZ.

[4]Department of Chemistry, University of Copenhagen, 2100 Copenhagen, Denmark

z.mukadam19@imperial.ac.uk
sihliu@dtu.dk
sbs@chem.ku.dk
yuxiang.zhou19@imperial.ac.uk
geokast@dtu.dk
m.p.ryan@imperial.ac.uk
m.titirici@imperial.ac.uk
i.stephens@imperial.ac.uk

Table of Contents

Computational Data	S3
NMR Spectroscopy	S4
XPS	S7
Electrochemical Data	S8
SEM and EDX	S10
Table of Reductive Amination Comparison	S11
References	S12

Computational Data

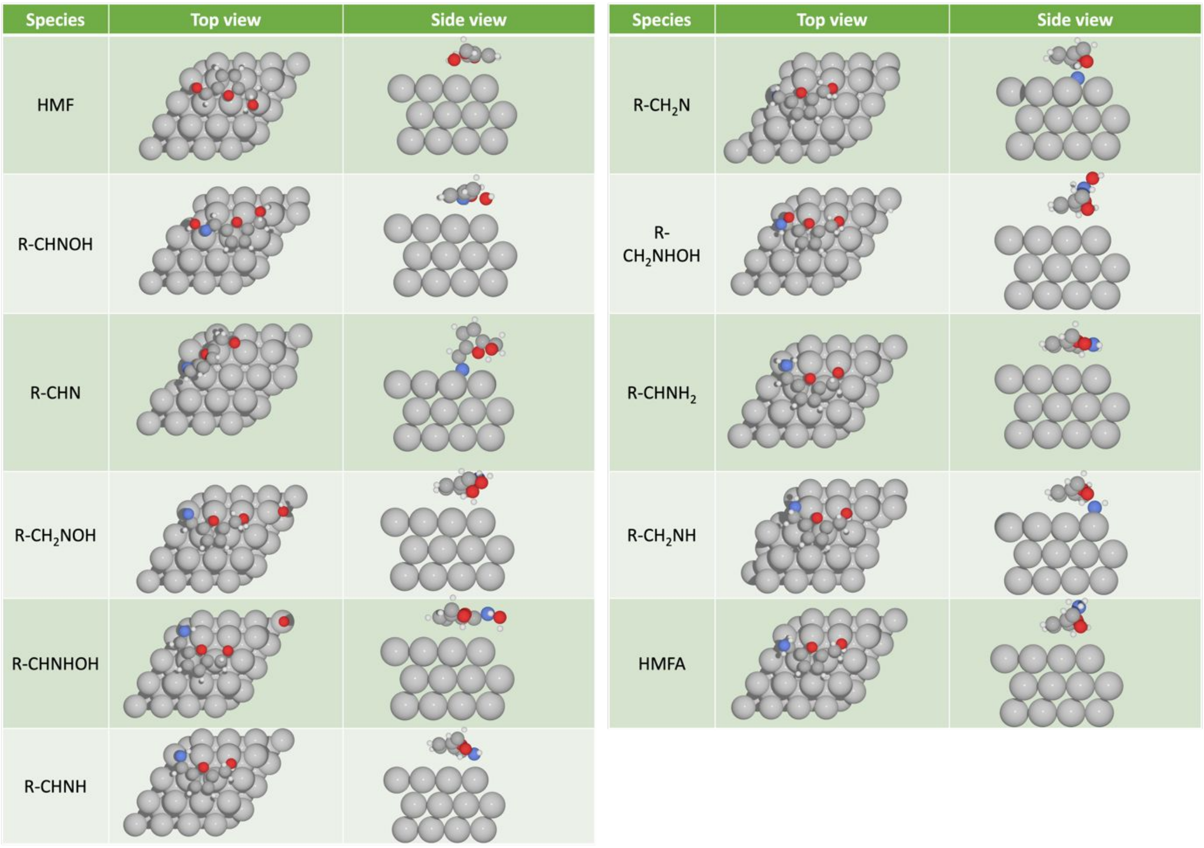

Figure S1. The optimized surface intermediates for HMF reductive amination over Ag(111).

NMR Spectroscopy

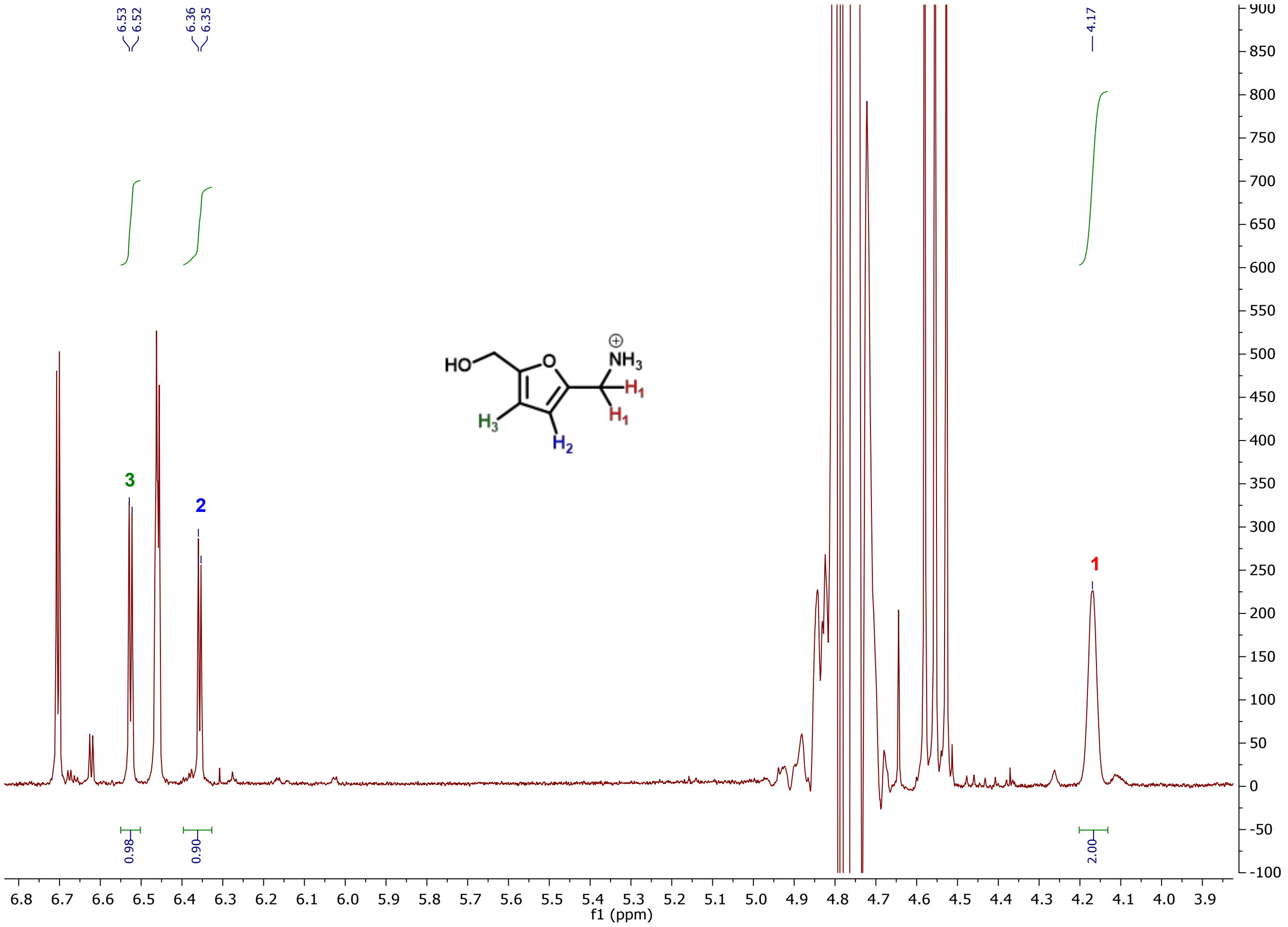

Figure S2. 400 MHz ^{1}H NMR spectra of HMFA produced after electrochemical reductive amination experiments. Peaks assigned using literature values for the same compound.[1] Chemical shifts between 4.5-5.0 ppm are from water peaks which were suppressed using NOESY. Consequently, chemical shifts from HMFA in this region are not assigned due to difficultly in distinguishing them from the water peaks. 5 mM DMSO was used as an internal standard to quantify the amount of HMFA.

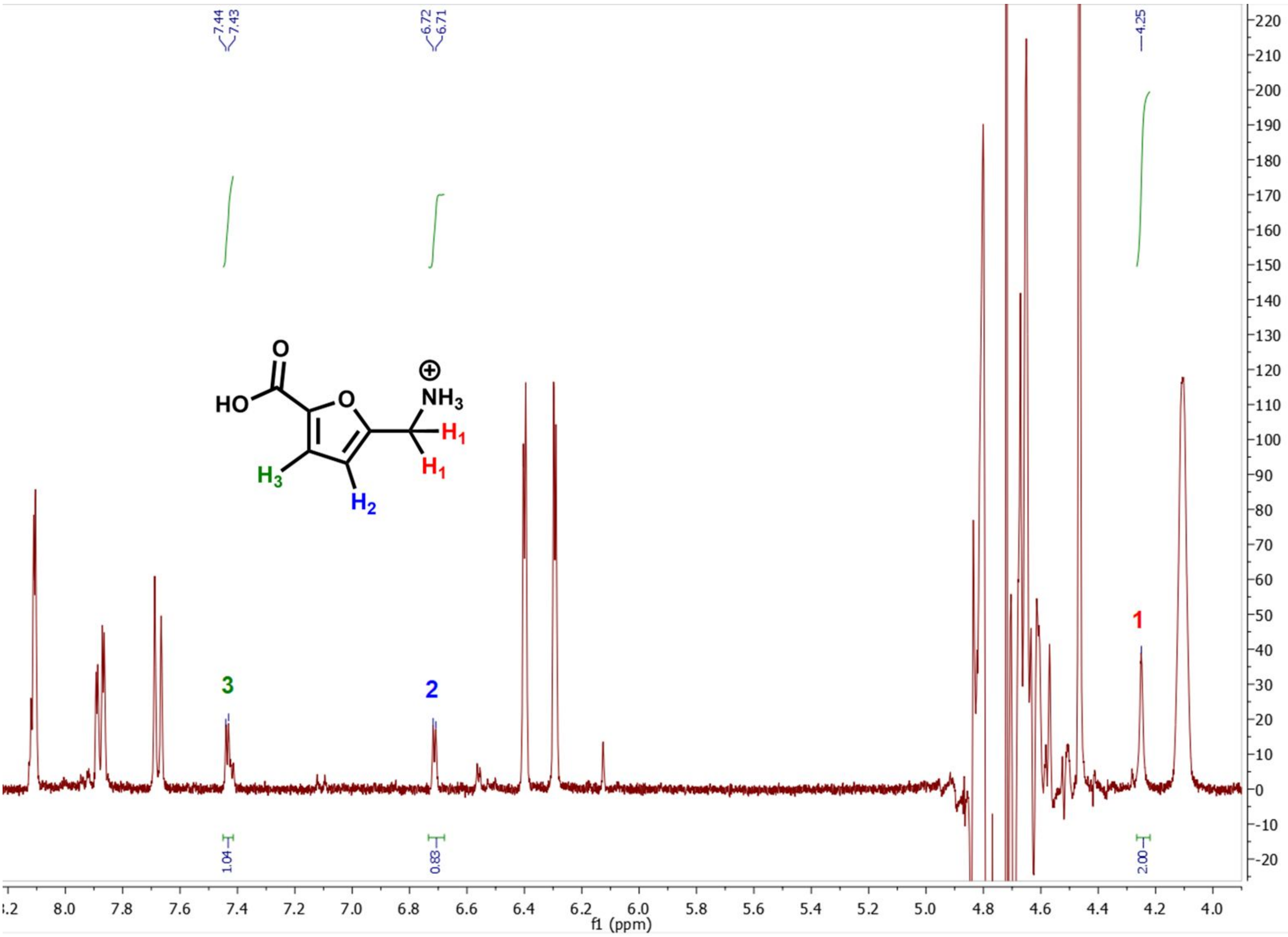

Figure S3. 400 MHz ^{1}H NMR spectra of AFCA after electrochemical synthesis. Peaks were assigned and confirmed using literature values for the same compound.[2] Chemical shifts between 4.6-5.0 ppm are from water peaks after NOESY water suppression techniques. 5 mM DMSO was used as an internal standard to quantify the amount of AFCA.

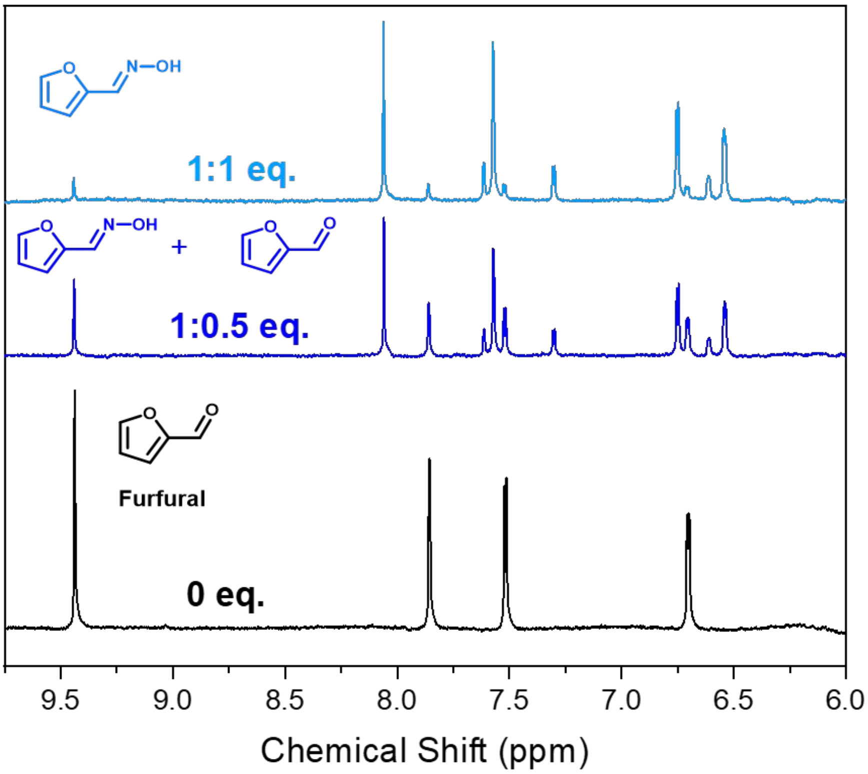

Figure S4. 400 MHz ^{1}H NMR of 20 mM furfural with different equivalents of NH_2OH in 0.1 M $HClO_4$ solution.

XPS

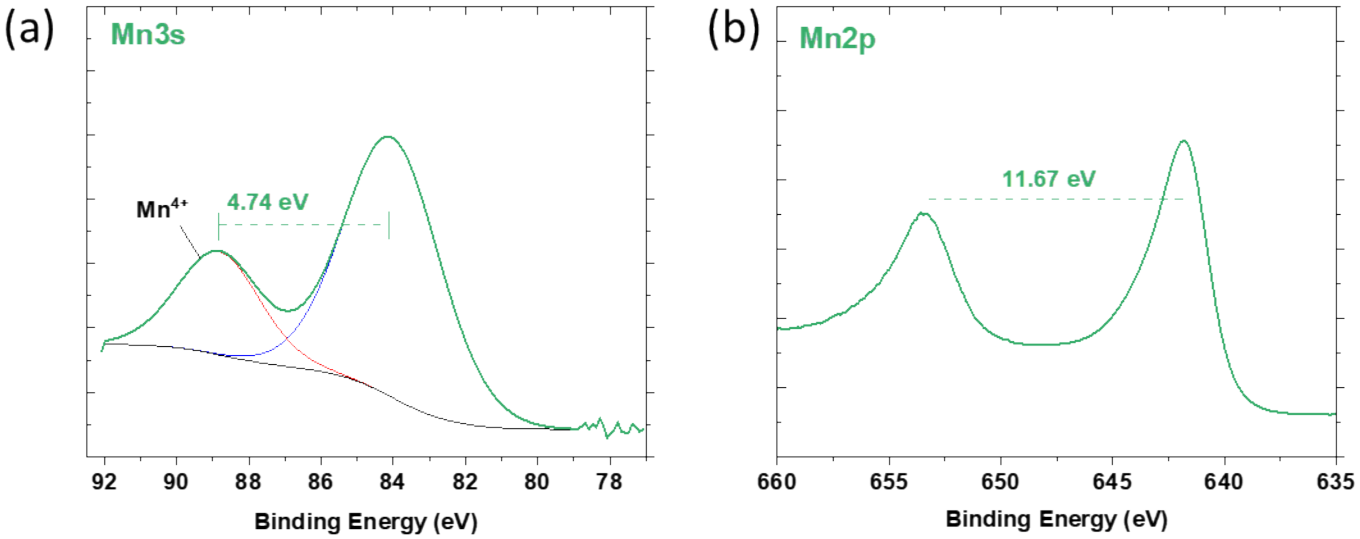

Figure S5. (a) Mn3s and (b) Mn2p core XPS spectra of sputtered manganese oxide (MnO_x) on Freudenberg H17 carbon paper.

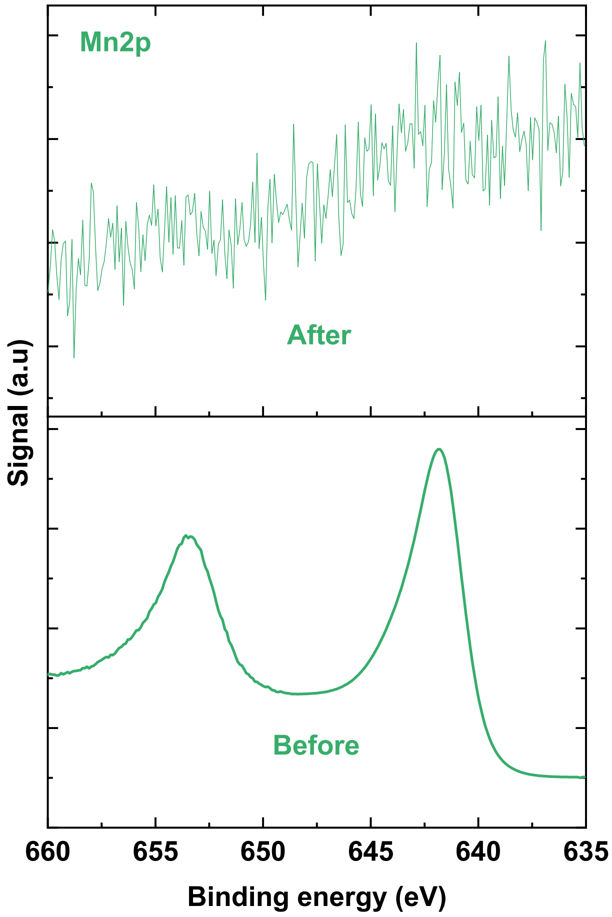

Figure S6. Mn2p core level XPS spectra of MnO_x anodes sputtered onto Freudenberg H17 carbon paper. Spectra taken before and after 3 h constant potential electrolysis at 1.60 V_{RHE} in 0.1 M $HClO_4$ (pH 1).

Electrochemical Data

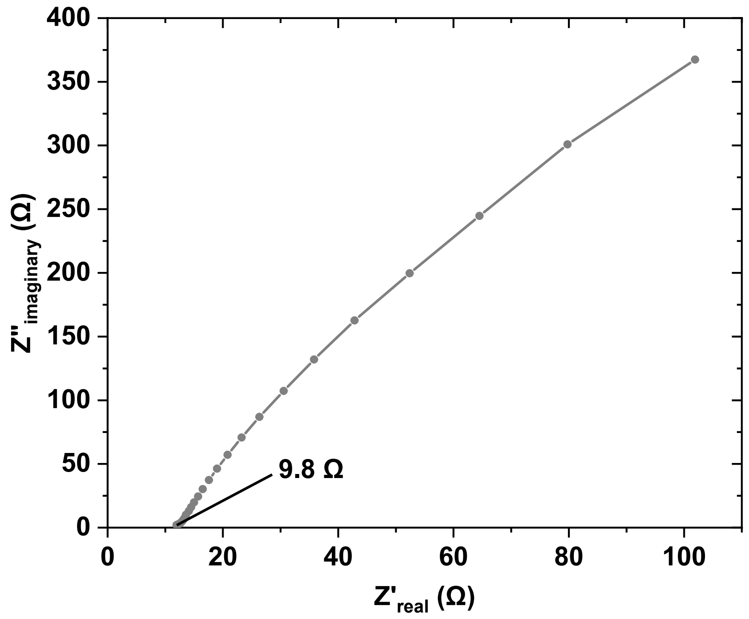

Figure S7. Electrochemical impedance spectroscopy of Ag electrode in a 0.1 M $HClO_4$ electrolyte (pH 1).

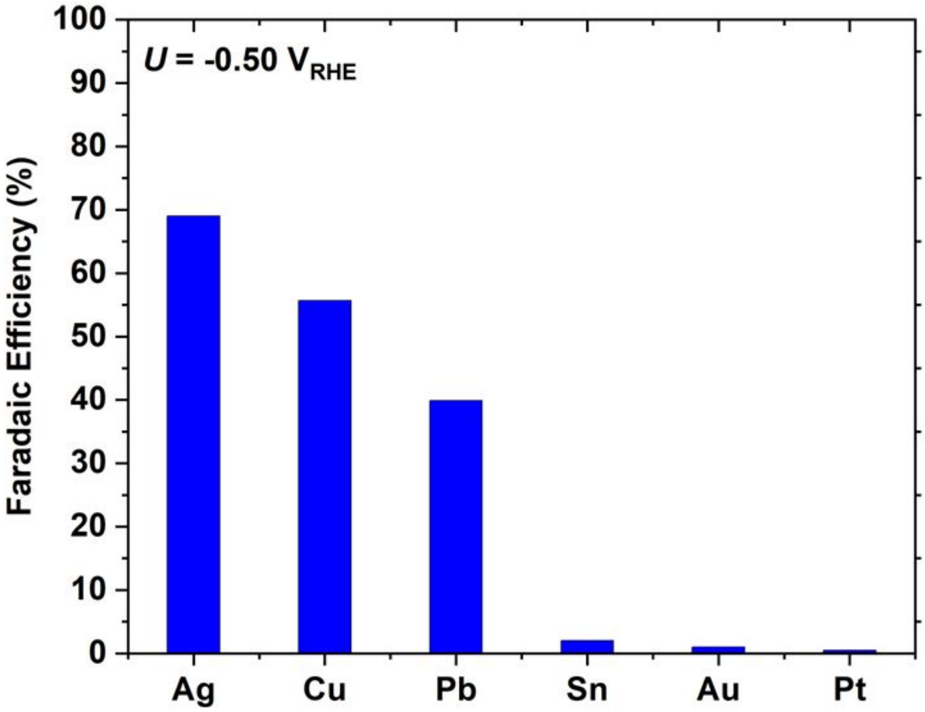

Figure S8. Faradaic efficiencies of tested metal electrodes for the electrochemical reductive amination of HMF into HMFA using NH_2OH. Reaction condition: 0.1 M $HClO_4$ electrolyte (pH 1), 10 mM HMF, 10 mM NH_2OH, potential was held at -0.50 V_{RHE} for each electrode for 3 h.

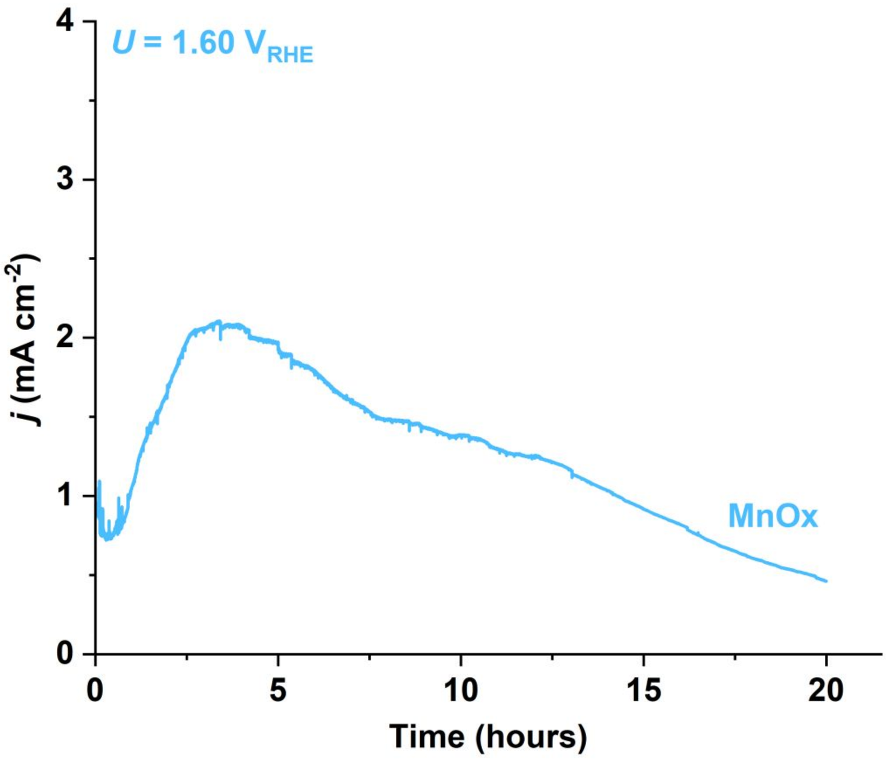

Figure S9. Chronoamperometry of MnO_x HMFA oxidation. Reaction conditions: 0.1 M $HClO_4$ electrolyte (pH 1), 10 mM HMFA, constant potential at 1.60 V_{RHE} for 20 hours.

SEM and EDX

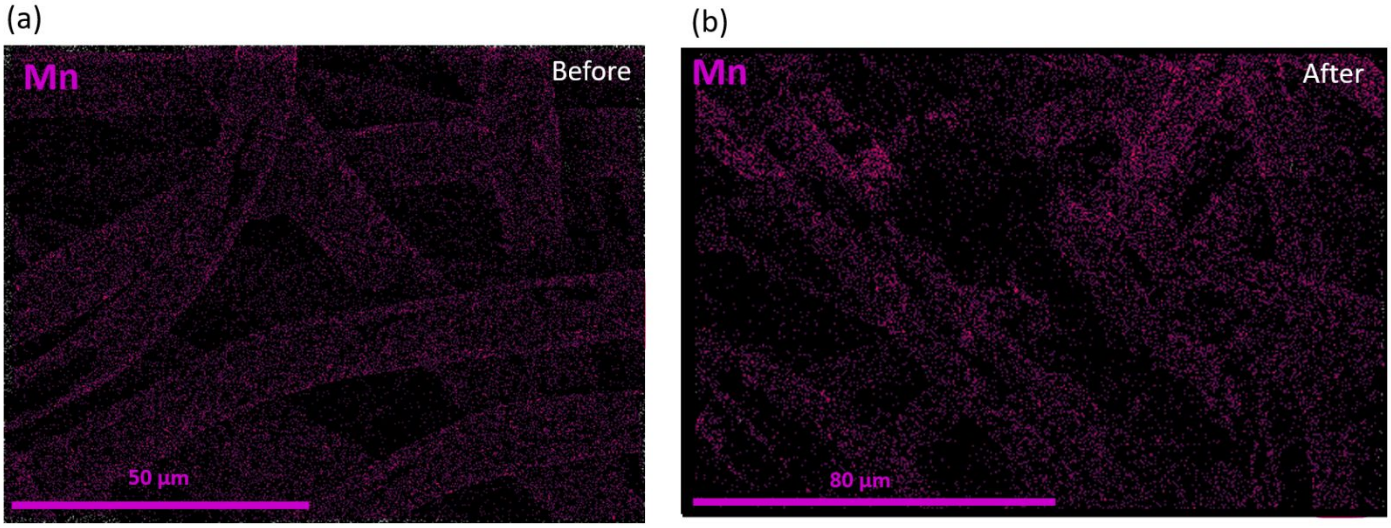

Figure S10. SEM and EDX images of MnO_x anodes before (a) and after (b) HMFA oxidation. Reaction conditions: 0.1 M $HClO_4$ electrolyte (pH 1), 10 mM HMFA, constant potential at 1.60 V_{RHE} for 20 hours.

Table of Reductive Amination Comparison

Catalyst	Substrate	Product	N source	Electrolyte	Potential	FE (%)	Ref.
TiO_2	Pyruvic acid	Alanine	NH_2OH	0.5 M H_2SO_4	2.0 V cell potential	75	3
TiO_2	Oxalic acid	Glycine	NH_2OH	0.5 M H_2SO_4	-0.7 V cell potential	28	4
TiO_2	Pyruvic acid	Alanine	NH_2OH	0.5 M H_2SO_4	-0.4 V_{RHE}	99	5
TiO_2	Pyruvic acid	Alanine	NH_3	1.5 M NH_3 buffer	-0.32 V_{RHE}	28	5
Ag	HMF	HMMAMF	CH_3NH_2	0.7 M CH_3NH_2 buffer	-1.1 $V_{Ag/AgCl}$	99	6
Cu	HMF	HMMAMF	CH3NH2	0.7 M CH3NH2 buffer	-1.2 $V_{Ag/AgCl}$	84	6
Ti-MOF	HMF	HEMF	ethanolamine	0.7 M ethanolamine buffer	-0.6 V_{RHE}	96	7
Ag	benzaldehyde	benzylamine	NH_3	1 M TBA-BF4	-1.36 V_{NHE}	80	8
Ag	HMF	HMFA	NH_2OH	0.1 M $HClO_4$	-0.50 V_{RHE}	72	This study

Table S1. Comparison of electrochemical amination reactions of various biomass molecules and reaction conditions. Abbreviations: FE – Faradaic efficiency, HMMAMF – 2-hydroxymethyl-5-(methylamino methyl) furan, MOF – metal-organic framework, HEMF – 2-hydroxymethyl-5-(ethanolamine methyl) furan, TBA-BF4 – tetrabutylammonium tetrafluoroborate.

References

1 A. Lancien, R. Wojcieszak, E. Cuvelier, M. Duban, P. Dhulster, S. Paul, F. Dumeignil, R. Froidevaux and E. Heuson, Hybrid Conversion of 5 -Hydroxymethylfurfural to 5 -Aminomethyl- 2 -furancarboxylic acid: Toward New Bio-sourced Polymers, *ChemCatChem*, 2021, **13**, 247–259.

2 A. Dunbabin, F. Subrizi, J. M. Ward, T. D. Sheppard and H. C. Hailes, Furfurylamines from biomass: Transaminase catalysed upgrading of furfurals, *Green Chem.*, 2017, **19**, 397–404.

3 T. Fukushima and M. Yamauchi, Electrosynthesis of amino acids from biomass-derivable acids on titanium dioxide, *Chem. Commun.*, 2019, **55**, 14721–14724.

4 T. Fukushima and M. Yamauchi, Electrosynthesis of glycine from bio-derivable oxalic acid, *J. Appl. Electrochem.*, 2021, **51**, 99–106.

5 C. Englezos, A. Raman, D. Jonker, N. A. Ramos-Delgado, M. Altomare, H. Gardeniers and A. Susarrey, Alanine Formation in a Zero-Gap Flow Cell and the Role of TiO 2 /Ti Electrocatalysts, *Chempluschem*, 2024, **89**, 1–11.

6 J. J. Roylance and K. S. Choi, Electrochemical reductive amination of furfural-based biomass intermediates, *Green Chem.*, 2016, **18**, 5412–5417.

7 M. Zhang, D. Hu, Y. Chen, Y. Jin, B. Liu, C. H. Lam and K. Yan, Electrocatalytic Reductive Amination and Simultaneous Oxidation of Biomass-Derived 5-Hydroxymethylfurfural, *Ind. Eng. Chem. Res.*, 2022, **61**, 1912–1919.

8 Z. J. Schiffer, M. Chung, K. Steinberg and K. Manthiram, Selective electrochemical reductive amination of benzaldehyde at heterogeneous metal surfaces, *Chem Catal.*, 2023, **3**, 100500.
